# Mixed Planting Can Improve Leaf Gas Exchange by Diversifying Plant Water Absorption Strategy

**DOI:** 10.1002/ece3.72875

**Published:** 2026-01-11

**Authors:** Xiao Liu, Meng Meng, Meijuan Zong, Hongkuan Hui, Yufei Xie, Simiao Wang

**Affiliations:** ^1^ School of Geography and Tourism Qilu Normal University Jinan China

**Keywords:** forest restoration, *Quercus acutissima*, *Robinia pseudoacacia*, season, warm temperate zone, water availability

## Abstract

Plants with diverse neighbors often exhibit significant variation in water absorption strategies, yet the responses of leaf gas exchange traits to water absorption strategies remain complex and uncertain. Therefore, we examined *Robinia pseudoacacia* and *Quercus acutissima* forests in the warm temperate zone, quantifying the water absorption strategies and leaf gas exchange traits across seasons to assess the impact of water absorption strategies dynamics. Our results demonstrate that mixed planting significantly enhanced the net photosynthetic rate compared to pure planting across all seasons, with a particularly pronounced effect for *Q. acutissima* . Furthermore, mixed planting promoted instantaneous water use efficiency in *R. pseudoacacia* while reducing transpiration and leaf midday water potential in *Q. acutissima* . The relationship between leaf gas exchange traits and water absorption strategies varies seasonally: in spring, a significant positive correlation was found between net photosynthetic rate and water absorption strategy; in summer, a similar correlation was observed between transpiration rate and water absorption strategy; in autumn, a significant positive correlation was noted between instantaneous water use efficiency and water absorption strategy, whereas a significant negative correlation was found between leaf midday water potential and water absorption strategy. These findings reveal that the seasonal responses of leaf gas exchange traits to water absorption strategies reflect key adaptive mechanisms of *R. pseudoacacia* and *Q. acutissima* . This study provides critical scientific guidance for optimizing forest restoration and management strategies, particularly in enhancing productivity and mitigating water competition in warm temperate zones.

## Graphical abstract

Our results show that mixed planting led to a high net photosynthetic rate, transpiration rate, and midday water potential; the correlation among leaf gas exchange traits and water absorption strategy was seasonal.

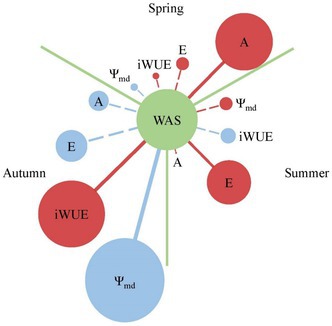

## 1 Introduction

One of the key components in global climate change is the significant alteration in precipitation patterns (Ge et al. 2017; Liu, Wang, et al. 2021). This phenomenon exacerbates the existing uneven distribution of temporal and spatial water in the warm temperate zone (Luan et al. 2011; Corlett 2016). The warm temperate zone has four distinct seasons, resulting in seasonal variation in precipitation. Seasonality in precipitation drives plants to be dependent on soil water and groundwater during drought periods, representing diverse plant water absorption strategies (Wright et al. 1992; Liu et al. 2020). To gain a deeper understanding of the response strategies of plants to warm temperate seasonal changes, it is urgent to study the seasonal differences in plant water absorption strategies (Ohte et al. 2003; Bailey 2009; Wang et al. 2010).

Plant water absorption strategy reflects plant water sources, describes plant water uptake ratio of rainwater, soil water, and groundwater (Hartmann and Trumbore 2016). It depends on the root form of the species, and it can be affected by seasons and neighbors (Liu et al. 2023). Deep‐rooted species have a diverse plant water absorption strategy; they could use groundwater, soil water, and rainwater selectively (David et al. 2007). While shallow‐rooted species have a single plant water absorption strategy, they get no access to groundwater but have strong competitiveness for soil water and rainwater (Schenk and Jackson 2002; Liu et al. 2023). Due to seasonality in the availability of soil water and rainwater, species may adopt different plant water absorption strategies to cope with the changing environment (Hoffmann et al. 2011).

Plants can actively respond to water availability by altering their leaf gas exchange traits (Liu, Zhang, et al. 2021). Drought could cause hydraulic failure, reduce photosynthetic rate and transpiration rate, leading to plant desiccation (Anderegg et al. 2015). Meanwhile, drought could also change plant water absorption strategy; it may make plant species get more use of groundwater (Hartmann and Trumbore 2016). Thus, water availability could not only affect leaf gas exchange traits but also affect plant water absorption strategy. A few researches have found that plants with diverse water absorption strategies may have higher leaf gas exchange traits than those with single water absorption strategy (Lönnqvist et al. 2023). However, further research is needed to deeply discuss the correlation between leaf gas exchange traits and plant water absorption strategy under changing water availability.

Forest plants always have their neighbors. “A single tree does not make a forest.” Ecologists point out that different forest complexes often show their unique adaptation when facing ecological challenges (Forrester and Bauhus 2016; Liu, Zhang, et al. 2021). Scientific management of mixed forests can help reduce competition for scarce water resources and improve leaf gas exchange traits (Lebourgeois et al. 2013; Pretzsch et al. 2013). Plant communities rich in species diversity, with different root architectures and distribution patterns, show a relatively low level of water competitiveness compared to pure forests (Schwendenmann et al. 2015; Blackman et al. 2019). However, when competing species have similar niches, the overlap of ecological niches may lead to more intense competition. Root competition and diverse plant water absorption strategies can significantly influence leaf gas exchange traits (Pretzsch et al. 2013; Grossiord et al. 2014). Plant roots distributed in different depths can minimize competition and optimize water use through temporal and spatial partitioning in mixed forests (Goldsmith et al. 2012; Barbeta and Peñuelas 2017). Therefore, whether mixed planting could improve leaf gas exchange traits should be further studied.

**FIGURE 1:**
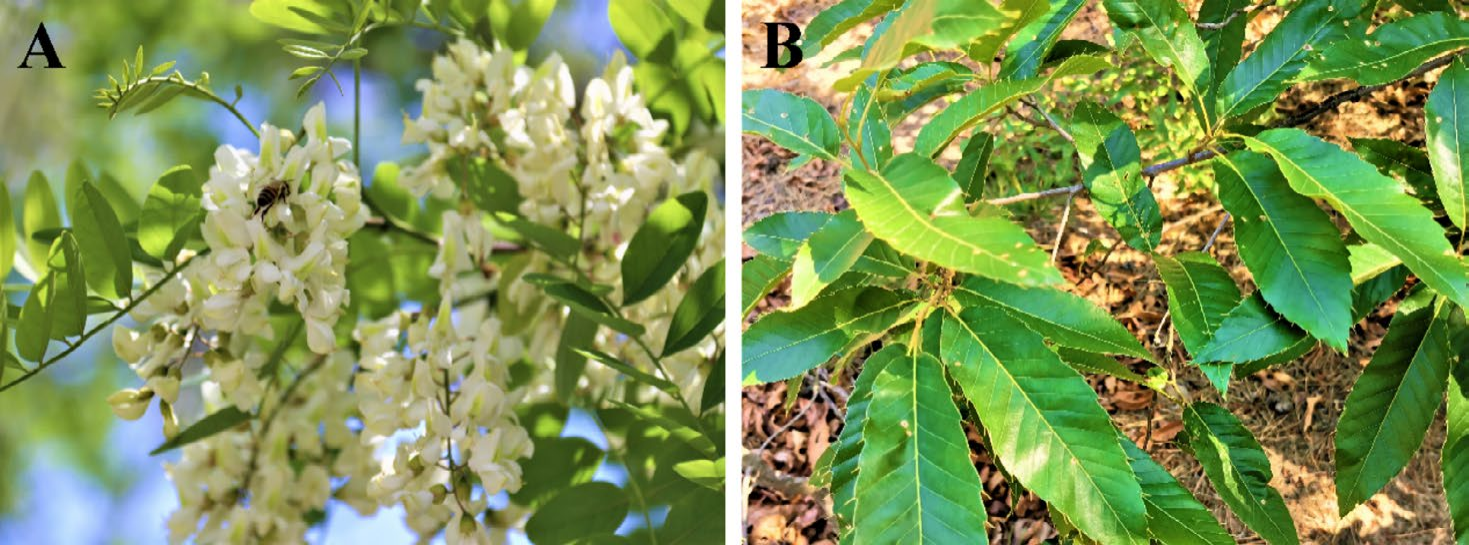
*R. pseudoacacia* (A) and *Q. acutissima* (B).

Temperate deciduous broadleaved forests are widely distributed in the warm temperate zone in North China, with shallow‐rooted *Robinia pseudoacacia* L. and deep‐rooted *Quercus acutissima* Carr. as the constructive species (Liu, Zhang, et al. 2021; Wang et al. 2021; Figure 1). The two trees are drought‐tolerant species and widely used in afforestation, vegetation restoration, and precise forest quality improvement projects (Moser et al. 2016; Liu et al. 2023), and an increasing number of studies centered on the leaf gas exchange traits and plant water absorption strategy under various habitats (Shao et al. 2024; Liu et al. 2025). However, there is still a lack of knowledge concerning the correlation between leaf gas exchange and plant water absorption strategy in different seasons. In order to discuss how leaf gas exchange traits respond to the changes in plant water absorption strategy, we designed a field experiment and hypothesized that (1) mixed planting may improve leaf gas exchange traits, and (2) the effects of plant water absorption strategy on leaf gas exchange traits may be seasonal.

## 2 Materials and Methods

### 2.1 Experimental Design

**FIGURE 2:**
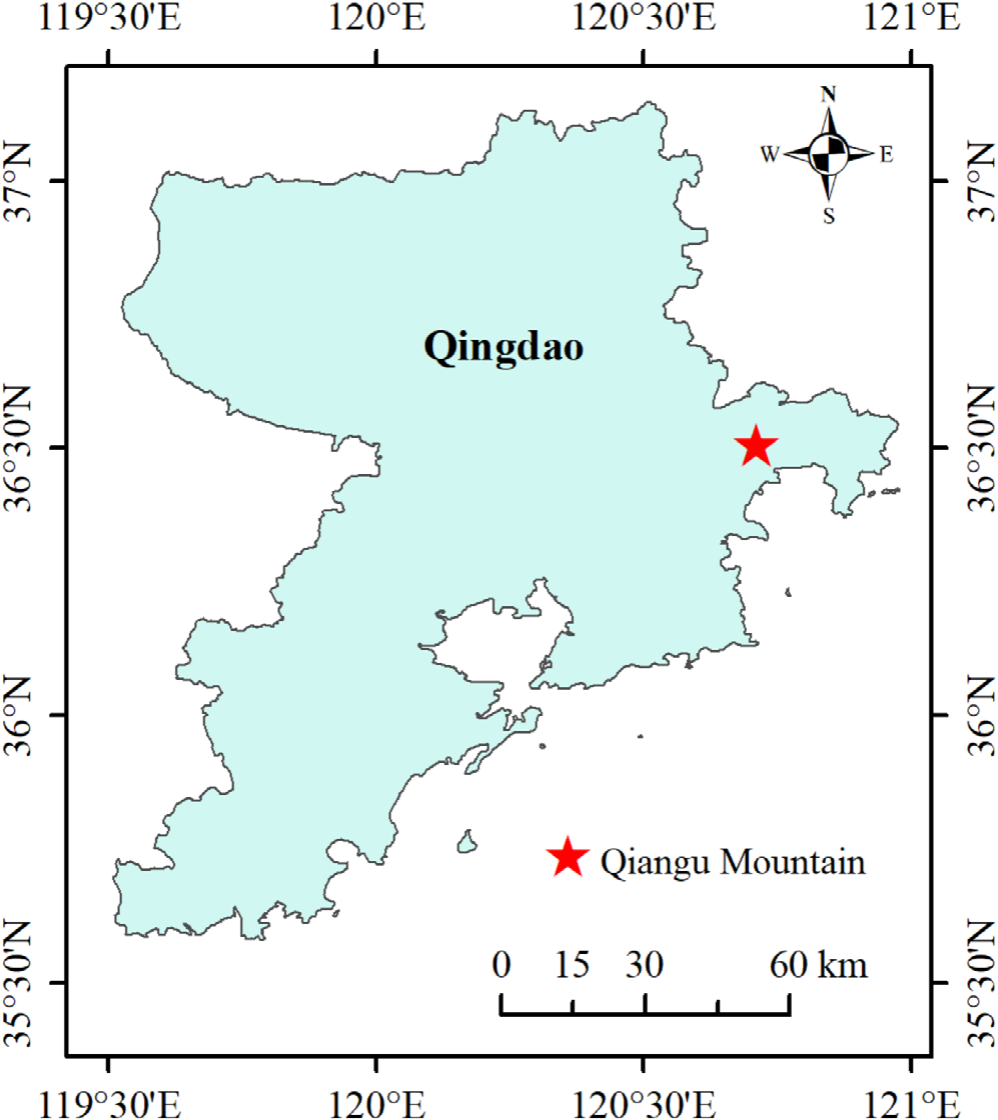
The location of Qiangu Mountain.

We conducted the field experiment at Qiangu Mountain in Jimo District, Qingdao City, Shandong Province, China (36.48° N, 120.72° E; Figure 2). The region is in North China with a warm temperate monsoon climate. After the afforestation in the 1950s, the mountain is dominated by *R. pseudoacacia* and *Q. acutissima* forests, which have been planted for over 70 years (Wang et al. 2023; Liu, Li, et al. 2024).

**FIGURE 3:**
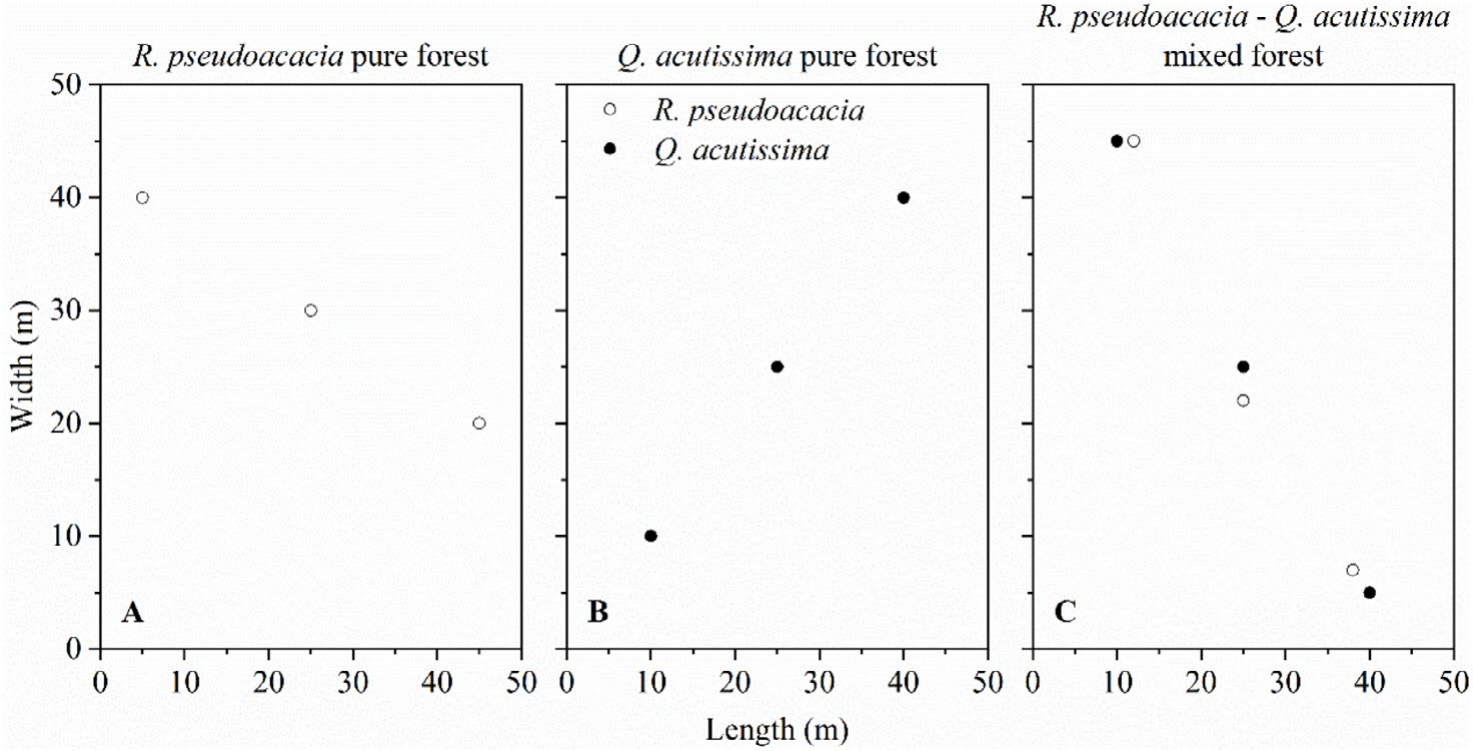
Coordinates of the 12 marked trees in *R. pseudoacacia* pure forest (A), *Q. acutissima* pure forest (B), and *R. pseudoacacia* – *Q. acutissima* mixed forest (C). Hollow circles, *R. pseudoacacia* ; solid circles, *Q. acutissima* .

At the same altitude of the southern slope in Qiangu Mountain, we selected *R. pseudoacacia* pure forest (RF), *Q. acutissima* pure forest (QF), and *R. pseudoacacia* – *Q. acutissima* mixed forest (MF) on the sunny side, the stand density of the forests is *c*. 1000 ha^−1^, and the distance between any two forests is greater than 20 m. We marked three *R. pseudoacacia* individuals (RP) and three *Q. acutissima* individuals (QP) respectively in their pure forest; the distance between any two marked trees was greater than 15 m. Meanwhile, in the *R. pseudoacacia* – *Q. acutissima* mixed forest, we marked three *Q. acutissima* individuals (QM) and three *R. pseudoacacia* (RM), the distance between any two marked trees was also greater than 15 m. In total, we had marked 12 individuals for our field experiment. The basic information of the forests and the marked trees are shown in Table 1, and the coordinates of these 12 marked trees are shown in Figure 3.

**TABLE 1:** Basic information of the forests and the marked trees, including forest type (FT), stand density (De, ha^−1^), species (Sp), plant height (*H*, m), diameter at breast height (DBH, cm).

FT	De	Sp	*H*	DBH
*R. pseudoacacia* pure forest	966	*R. pseudoacacia*	17.5	23.3
		*R. pseudoacacia*	18.0	22.8
		*R. pseudoacacia*	18.0	23.1
*Q. acutissima* pure forest	990	*Q. acutissima*	17.5	22.7
		*Q. acutissima*	18.0	22.4
		*Q. acutissima*	17.5	23.7
*R. pseudoacacia* – *Q. acutissima* mixed forest	1020	*R. pseudoacacia*	18.0	23.5
		*R. pseudoacacia*	17.5	22.8
		*R. pseudoacacia*	18.0	23.6
		*Q. acutissima*	18.0	23.2
		*Q. acutissima*	18.0	22.5
		*Q. acutissima*	17.5	22.9

We conducted the experiment on April 26th (spring), July 18th (summer), and October 14th (Autumn), 2023; monthly average temperature and monthly average precipitation of the sampling months are shown in Table 2. Sampling dates were the second day after the rain stopped to ensure similar soil water availability.

**TABLE 2:** Monthly average temperature (MAT) and monthly average precipitation (MAP) of the sampling months.

Seasons	MAT (°C)	MAP (mm)
April	14.83	24
July	28.45	290
October	11.62	31

On each sampling date: first, 4–6 mature branches from each of the 12 individuals were cut down with bark removed. Then, 0.2‐ to 0.3‐m‐deep soil was collected next to the marked individuals. Besides, groundwater was collected from the 5‐m‐deep sealed well near our plot. Furthermore, rainwater was collected with rain gauges on April 24th, July 16th, and October 12th before the rain stopped. Three replicates were set for rainwater, soil water, and groundwater in each season. Once the collection was completed, plant samples and environmental samples were immediately loaded into 50 mL covered centrifuge tubes, put into an ice box, and brought to the laboratory (Pérez‐Harguindeguy et al. 2016; Liu et al. 2023). Totally, 36 plant samples and 45 environmental samples were collected for plant water absorption strategy quantification.

Then, we selected and cut down three sunny, intact, healthy mature branches in the middle of the canopy from each of the 12 marked individuals; the cut branches were immediately soaked in a bucket of degassed water. Fully expanded and healthy leaves on the branches were picked for gas exchange traits and midday water potential measurements. We also sampled soil from the three plots for soil nitrogen content determination.

### 2.2 Leaf Gas Exchange Traits

Leaf gas exchange traits include net photosynthetic rate (*A*, μmol m^−2^ s^−1^), transpiration rate (*E*, mmol m^−2^ s^−1^), instantaneous water use efficiency (iWUE, ‰), and leaf midday water potential (Ψ_md_, MPa). The picked leaves of the marked trees were chosen for measurements on sampling dates with an infrared gas analysis system (Li‐6800, Li‐Cor, Lincoln, NE, USA). The photosynthetic photo flux density was supplied by an external light emitting diode light and it was set at 1000 μmol m^−2^ s^−1^ according to Liu, Yi, et al. (2024). The measurements of gas exchange traits were conducted at 9:00 to 11:00 on each sampling day. During the measurement, temperature, relative humidity, and CO_2_ concentration inside the chamber were controlled at 28°C, 50%, and 400 ppm, respectively (Wang et al. 2020; Liu, Wang, et al. 2021). To improve accuracy and precision, measurements were finished within 10 min after samples had been cut down. Instantaneous water use efficiency was calculated as the quotient of net photosynthetic rate and transpiration rate.

Leaf midday water potential was measured between 11:00 and 13:00 in a pressure chamber (1505D‐EXP; PMS Instrument Company, Albany, OR, USA).

### 2.3 Plant Water Absorption Strategy

Samples for plant water absorption strategy (WAS) quantification were pretreated. Rainwater and groundwater were stored after filtering. Water from branches and soil was stored after cryogenic vacuum distillation (West et al. 2006). All water samples were stored in 10 mL centrifuge tubes. Isotope ratios were determined by an isotope ratio mass spectrometer (Mat 253 plus, Thermo Fisher Scientific, Effeltrich, Germany), and standard groundwater was used as the standard water sample. Based on the isotope ratios, plant water absorption strategy was quantified by IsoSource model and geometric mean (Phillips and Gregg 2003; Liu et al. 2023).

### 2.4 Soil Nitrogen Content

We randomly selected 10 points in each forest, then collected 0.2‐ to 0.3‐m‐deep soil from each point. The soil samples we used for soil nitrogen content measurement were mixed by soil from the 10 random points. We took three soil samples for one forest in each season. All samples were oven‐dried for 48 h at 80°C. Soil nitrogen content (‰) was determined by an automatic Kjeldahl apparatus (K9860, Hanon, Jinan, China).

### 2.5 Statistics

Data were checked for normality (Shapiro–Wilk test) and homogeneity (Levene test). Plant water absorption strategy was quantified by IsoSource model (Liu et al. 2023). Linear mixed effect models were fitted to detect the fixed effects of species and planting methods, and the random effects of seasons on leaf gas exchange traits, plant water absorption strategy, and soil nitrogen content. One‐way analysis of variance (One‐way ANOVA) followed by Duncan's multiple comparison test was used to detect the differences in leaf gas exchange traits among all groups within seasons and to detect the differences in soil nitrogen content among all groups. Spearman correlation analysis was conducted to detect the correlation between leaf gas exchange traits and plant water absorption strategy. The critical value *α* was set as 0.05. All statistical analyses were performed using the SPSS 26 software package (SPSS Inc., Chicago, IL, USA), and all figures were drawn using OriginPro 2024 SR1 (Originlab Co., Northampton, MA, USA).

## 3 Results

### 3.1 Effects of Planting Method and Season

There were significant interspecific differences in all traits, but the fixed effect of planting method was only significant for A and SNC. The random effect of season was significant on all traits. Besides, the interactions were also significant on all traits except the interactions of species and planting method on A, WAS, SNC, and the interactions of all the effects on A, SNC (Table 3).

**TABLE 3:** Effects of species (Sp), planting method (PM), and season (Se) on net photosynthetic rate (*A*, μmol m^−2^ s^−1^), transpiration rate (*E*, mmol m^−2^ s^−1^), instantaneous water use efficiency (iWUE, ‰), leaf midday water potential (Ψ_md_, MPa), water absorption strategy (WAS), and soil nitrogen content (SNC, ‰).

Traits	Sp	PM	Se	Sp × PM	Sp × Se	PM × Se	Sp × PM × Se
*A*	26.45**	6.85*	241.86**	0.69	38.36**	7.17**	2.00
*E*	91.45**	0.42	237.85**	20.70**	216.69**	5.09*	9.79*
iWUE	63.54**	0.26	72.51**	23.49**	57.93**	12.84**	7.83**
Ψ_md_	17.47**	1.08	44.36**	10.69*	34.84**	10.32**	7.24**
WAS	5.40*	8.27*	32.09**	4.27	10.53**	66.20**	5.45*
SNC	220.96**	59.67**	19.03**	0.00	4.63*	4.96*	0.00

*Note:* Data stand for *F* values of the mixed effect model.

* *p* < 0.05.

** *p* < 0.01.

### 3.2 Leaf Gas Exchange Traits

#### 3.2.1 Net Photosynthetic Rate

**FIGURE 4:**
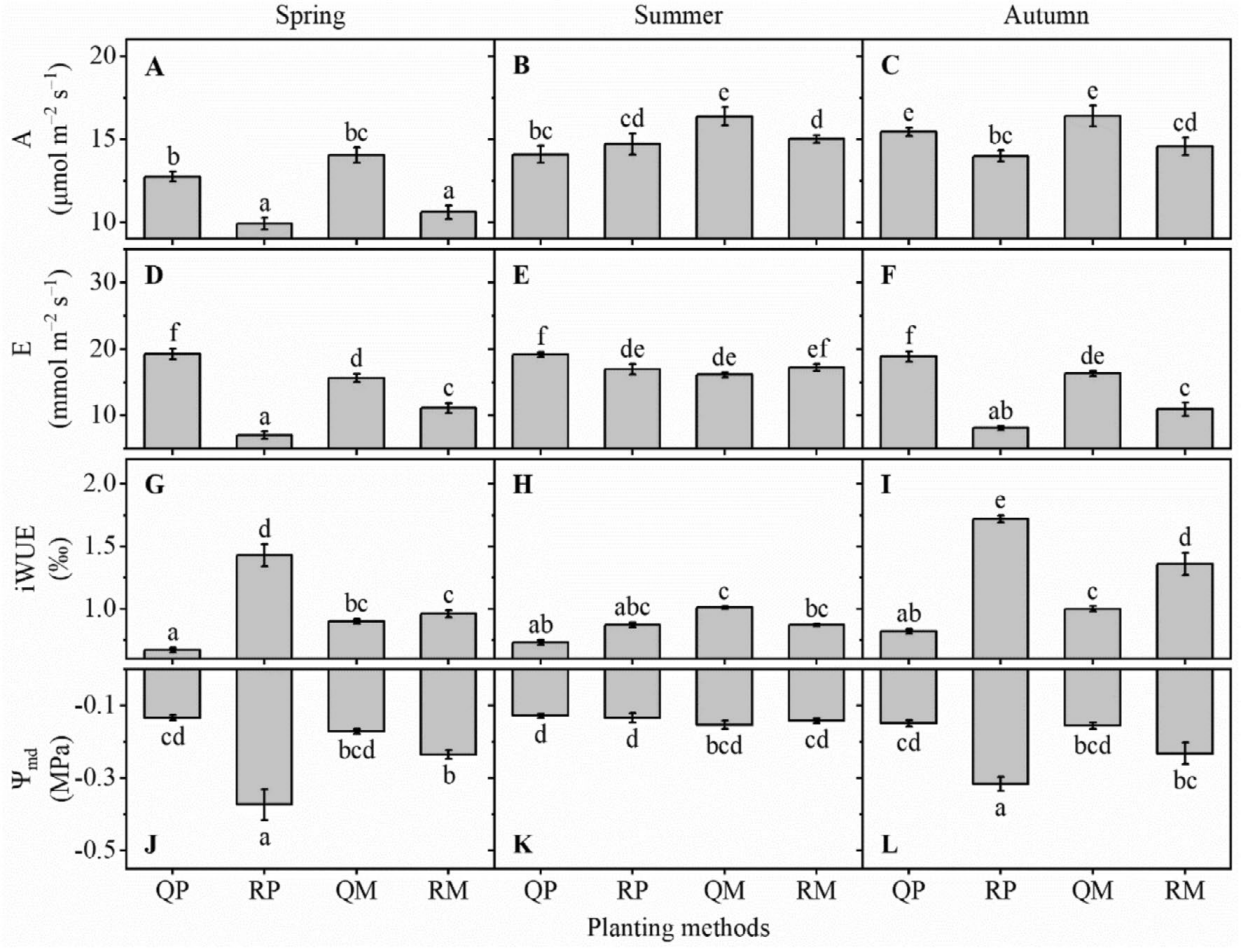
Leaf functional traits of *R. pseudoacacia* and *Q. acutissima* in spring (A, D, G, J), summer (B, E, H, K), and autumn (C, F, I, L), under different planting methods. *A*, net photosynthetic rate; *E*, transpiration rate; iWUE, instantaneous water use efficiency; Ψ_md_, midday water potential; QP, *Q. acutissima* in pure planting; RP, *R. pseudoacacia* in pure planting; QM, *Q. acutissima* in mixed planting; RM, *R. pseudoacacia* in mixed planting. Data stand for mean ± 1 SE, *n* = 3. Different letters represent significant differences among all groups, *p* < 0.05.

In spring and autumn, A of and *Q. acutissima* was significantly higher than that of *R. pseudoacacia* (Figure 4A,C). In summer, all groups had high A, and A of QM was higher than any other group (Figure 4B).

#### 3.2.2 Transpiration Rate

In spring and autumn, E of QP was the highest among all groups, while E of RP was lowest among all groups (Figure 4D,F). In summer, all groups had high E (Figure 4E).

#### 3.2.3 Instantaneous Water Use Efficiency

In spring and autumn, iWUE of RP was the highest among all groups, while iWUE of QP was the lowest among all groups (Figure 4G,I). In summer, iWUE of QM was significantly higher than that of QP (Figure 4H).

#### 3.2.4 Midday Water Potential

In spring and autumn, Ψ_md_ of QP was significantly higher than RP while the difference between QM and RM was not obvious (Figure 4J,L). In summer, all groups had high Ψ_md_ (Figure 4K).

### 3.3 Correlation Between Leaf Gas Exchange Traits and Plant Water Absorption Strategy

**FIGURE 5:**
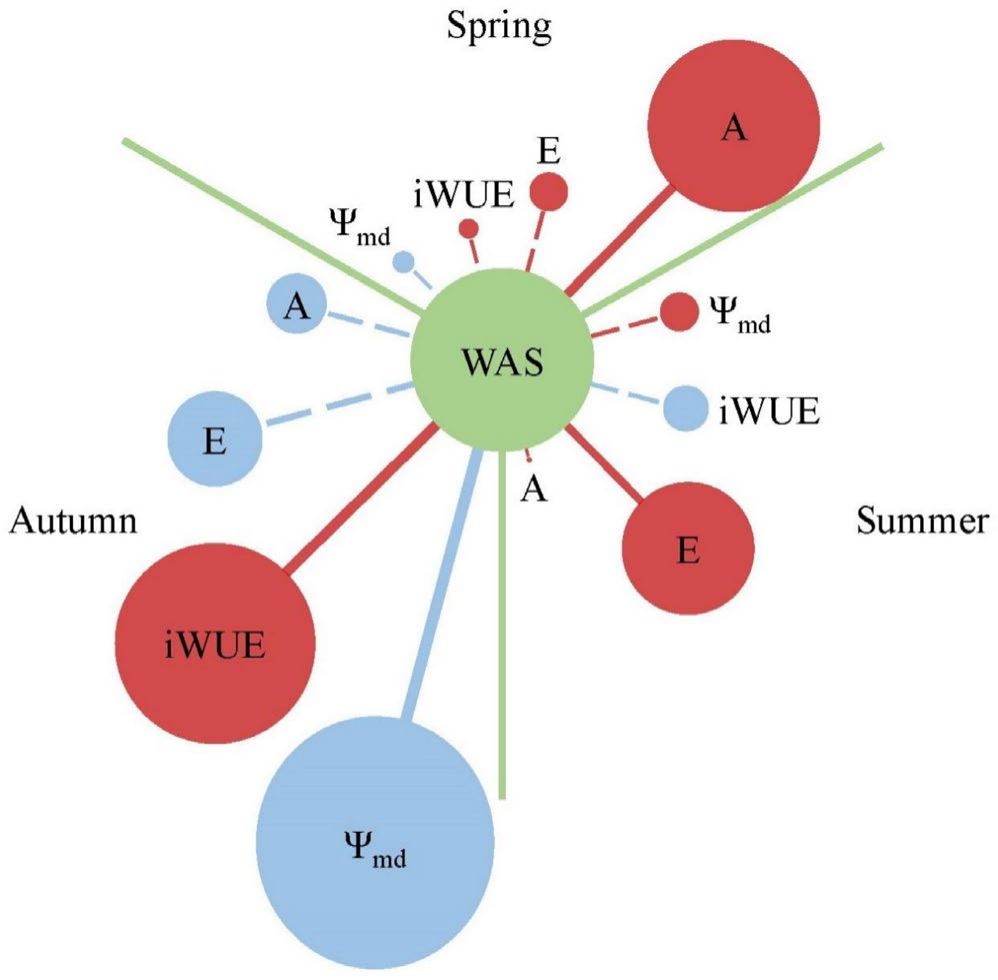
Spearman correlation among leaf functional traits and plant water absorption strategy (WAS) in different seasons. *A*, net photosynthetic rate; *E*, transpiration rate; iWUE, instantaneous water use efficiency; Ψ_md_, midday water potential. Red and blue circles indicate positive and negative correlation, respectively. The relative size of the circles stands for the relative strength of correlation. Solid and dash lines represent significance (*p* < 0.05) and insignificance, respectively.

In spring, A was significantly positive correlated to WAS, while E, iWUE, and Ψ_md_ had no obvious correlation to WAS. In summer, E was significantly positive correlated to WAS, while A, iWUE, and Ψ_md_ had no obvious correlation to WAS. In autumn, iWUE was significantly positive correlated to WAS, and Ψ_md_ was significantly positive correlated to WAS, while A and E had no obvious correlation to WAS (Figure 5).

### 3.4 Soil Nitrogen Content

**FIGURE 6:**
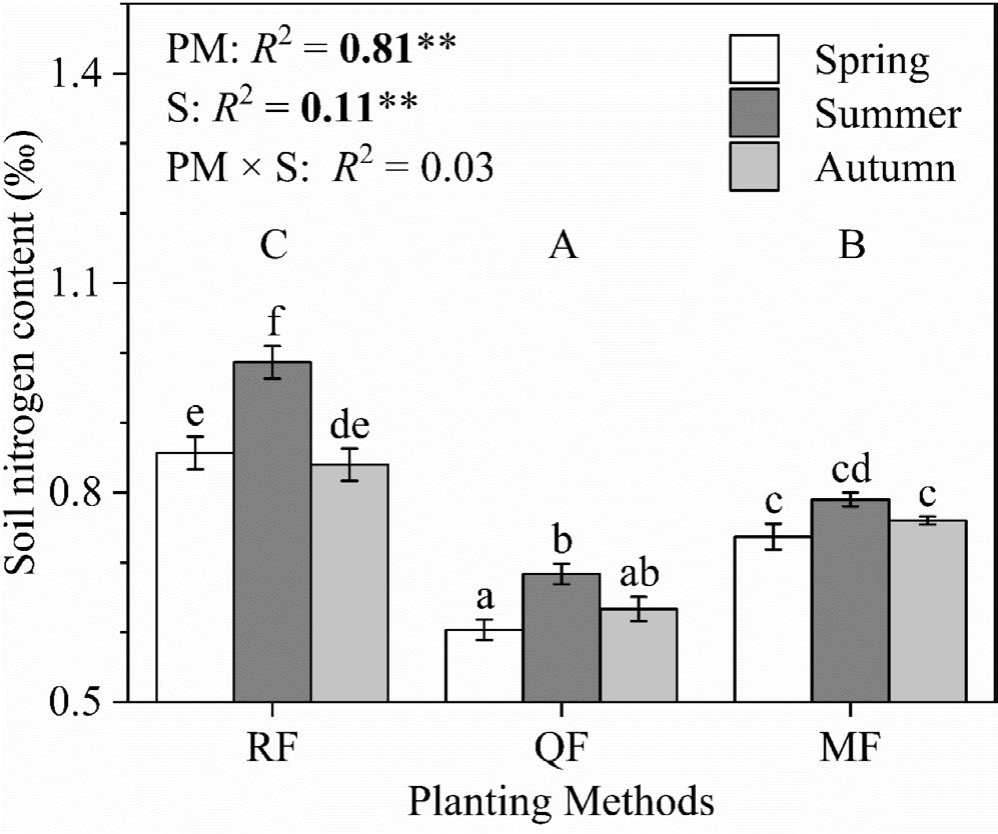
Soil nitrogen content of the *R. pseudoacacia* pure forest (RF), the *Q. acutissima* pure forest (QF), and the *R. pseudoacacia* – *Q. acutissima* mixed forest (MF). The proportion of explained variation (*R* ^2^) of planting method (PM), season (S), and their interaction on soil nitrogen content is shown, **p < 0.01. Different uppercase letters indicate significances among planting methods while different lowercase letters indicate significances among groups, *p* < 0.05.

Soil nitrogen content of MF was significantly higher than that of QF and it was significantly lower than that of RF regardless of the season. Planting method explained 81% of the changes in soil nitrogen content and season explained 11%. The total explanation of planting methods and season to soil nitrogen content was 95% (Figure 6).

## 4 Discussion

### 4.1 Mixed Planting Improved Leaf Gas Exchange Traits

Our results indicate that mixed planting improved leaf gas exchange, which is consistent with our Hypothesis (1). Ecologists point out that different forest complexes often show their unique adaptation when facing ecological challenges (Forrester and Bauhus 2016; Liu et al. 2020). In mixed forest, nitrogen‐fixing plant *R. pseudoacacia* can increase the soil nitrogen content under nitrogen fixation (Figure 6), improving soil nutrition availability (Brooker et al. 2008; Liang et al. 2016); furthermore, deep‐rooted species *Q. acutissima* can improve the soil water availability under hydraulic lifting (Liu et al. 2023), achieving soil water redistribution (Sekiya and Yano 2008; Prieto et al. 2012). Specifically, mixed planting could enable *Q. acutissima* to obtain more nutrition while it could also enable *R. pseudoacacia* to obtain enough water. That may be the reason why mixed planting led to higher net photosynthetic rate than pure planting in all seasons, especially for *Q. acutissima* (Figure 4A–C). Although *Q. acutissima* could improve the soil water availability for *R. pseudoacacia* , the presence of *R. pseudoacacia* may intensify shallow soil water competition, especially when water may be the limiting factor (Grossiord et al. 2014; Muys et al. 2016). Thus, mixed planting decreased transpiration rate and midday water potential of *Q. acutissima* , while it increased those of *R. pseudoacacia* in spring and autumn (Figure 4D–FJ–L).

Besides, mixed planting increased instantaneous water use efficiency of *Q. acutissima* , while it decreased that of *R. pseudoacacia* (Figure 4G–I). From the perspective of gas exchange, soil nutrition availability increased by *R. pseudoacacia* brought greater influence to *Q. acutissima* than soil water competition intensified by *R. pseudoacacia* (Brooker et al. 2008). Nutrition may be the limiting factor for photosynthesis of *Q. acutissima* (Forrester et al. 2005; Liang et al. 2016). While soil water availability increased by *Q. acutissima* did not improve the net photosynthetic rate of *R. pseudoacacia* , but caused it to squander water instead, resulting in a reduction in instantaneous water use efficiency. According to our results, water may not be the key factor for the gas exchange of *R. pseudoacacia* in summer (Prieto et al. 2012; Craine et al. 2013).

### 4.2 Correlation Between Leaf Gas Exchange Traits and Plant Water Absorption Strategy Was Seasonal

Changes in the correlation between leaf gas exchange traits and plant water absorption strategy indicate that plants may adopt diverse strategies to adapt to seasonal variation, which supports our Hypothesis (2) (Figure 5). Plant photosynthetic rate has a strong correlation to soil water and nutrition availability (Brooker et al. 2008, Liang et al. 2016). In spring, plants required more photosynthetic products for stem and leaf construction, but in the warm temperate zone, spring always lacks water, which was the limiting factor for plant photosynthesis and morphogenesis. Thus, the significant positive correlation between net photosynthetic rate and plant water absorption strategy may be beneficial for maintaining net photosynthetic rate at a high level during spring drought (Craine et al. 2013; Muys et al. 2016). With the increase in precipitation, water was no longer a limiting factor when summer came. At this time, diverse plant water absorption strategies had not only increased net photosynthetic rate but also led to plants squandering water, manifested as a significant increase in transpiration rate, especially in *R. pseudoacacia* (Forrester et al. 2005; Prieto et al. 2012). This phenomenon may be the reason why the more diverse plant water absorption strategies were, the larger transpiration rate plants had (Richards et al. 2010). In autumn, plant water absorption strategy had little correlation to gas exchange traits, with the decrease of precipitation, water became the limiting factor. According to our result, diverse plant water absorption strategy had a significant correlation to instantaneous water use efficiency and leaf midday water potential (Brooker et al. 2008; Craine et al. 2013). The improvement in instantaneous water use efficiency had increased net photosynthetic rate, at the cost of a decrease in leaf midday water potential. In autumn, diverse plant water absorption strategy may improve plant productivity, but intensify interspecific water competition (Liang et al. 2016).

In this research, we considered seasons as a whole, qualitative variable and only studied two constructive species of temperate deciduous broadleaved forests. Future researches could focus on the effect of multiple ecological factors from the perspective of plant species diversity through more detailed experimental designs to obtain more accurate and precise results.

## 5 Conclusion

Our results find that (1) mixed planting led to a high net photosynthetic rate, transpiration rate, and leaf midday water potential; (2) the correlation among leaf gas exchange traits and plant water absorption strategy was seasonal. Our research clarifies that mixed planting may improve leaf gas exchange, at a cost of intensifying shallow soil water competition. While seasonal responses of leaf gas exchange traits to plant water absorption strategy reflect the adaptation strategies of *R. pseudoacacia* and *Q. acutissima* in the warm temperate zone. This study provides critical scientific guidance for optimizing forest restoration and management strategies, particularly in enhancing productivity and mitigating water competition in warm temperate zones.

## Author Contributions

**Xiao Liu:** conceptualization (lead), funding acquisition (lead), project administration (lead), supervision (lead), writing – original draft (lead), writing – review and editing (lead). **Meng Meng:** data curation (equal), investigation (equal), methodology (equal). **Meijuan Zong:** conceptualization (supporting), funding acquisition (supporting), project administration (supporting), supervision (supporting), validation (lead), visualization (lead). **Hongkuan Hui:** data curation (equal), investigation (equal), methodology (equal). **Yufei Xie:** formal analysis (equal), resources (equal), software (equal). **Simiao Wang:** formal analysis (equal), resources (equal), software (equal).

## Funding

This work was supported by the Natural Science Foundation of Shandong Province (ZR2023QC238), the Shandong Province Social Science Planning Research Project (25CJYJ09), and the Fundamental Research Funds of Qilu Normal University (107002001365001).

## Conflicts of Interest

The authors declare no conflicts of interest.

## Supporting information


**Data S1:** ece372875‐sup‐0001‐Supinfo.xlsx.

## Data Availability

All the required data are uploaded as Supporting Information.
